# Activated Human Pancreatic Stellate Cells SignatureCommunication in Type 1 Diabetes

**DOI:** 10.21203/rs.3.rs-8704281/v1

**Published:** 2026-02-04

**Authors:** Gongxin Yu, Alejandra M Petrilli, Carley Glass, Yury Nunez Lopez, Richard E Pratley, Anna Casu

**Affiliations:** AdventHealth Research Institute; AdventHealth Research Institute; AdventHealth Research Institute; AdventHealth Research Institute; AdventHealth Research Institute; AdventHealth Research Institute

**Keywords:** Single-cell RNA sequencing, CellChat, type 1 diabetes, pancreatic stellate cells

## Abstract

**Background:**

Type 1 diabetes (T1D) is an autoimmune disease resulting in the destruction of pancreatic β-cells leading to insulin deficiency and hyperglycemia. Single cell transcriptomic analysis of human islets demonstrated profound β-cell changes and revealed heterogeneity in endocrine and exocrine cells in T1D. Pancreatic stellate cells (PSCs), the resident mesenchymal cells of the pancreas, regulate extracellular matrix homeostasis and drive fibrosis in aging, pancreatitis, and pancreatic cancer. By secreting cytokines and growth factors, PSCs contribute to local immunity and inflammation that affect pancreatic exocrine and endocrine functions. However, cell-cell communication from single cell transcriptomics analyzing the role of PSCs in T1D has not been explored.

**Methods:**

We analyzed single-cell RNA sequencing data from human pancreatic islets of 20 donors with and without T1D from the Human Pancreas Analysis Program database using the CellChat R package, focusing on activated-PSCs (aPSCs) signaling pathways. In addition, we performed aPSCs differential expression gene and gene set enrichment analyses.

**Results:**

CellChat analysis revealed aPSCs demonstrated major changes increasing the number and strength of cellular communications in T1D compared to control pancreata. Signaling pathways upregulated in cell-to-cell communication involving aPSCs include TGFB, FGF, CXCL, ANGPTL, and NGF, and their respective ligands TGFB1/3, FGF7, CXCL12, ANGPTL4 and NGF. In contrast, PTN signaling from aPSCs was blunted in T1D.

**Conclusions:**

Our study revealed novel intercellular communication signatures involving aPSCs in T1D. Identification of the changes in cellular communication between aPSCs and other cells in T1D suggest a role in T1D pathogenesis or progression which might lead to the development of novel therapeutics.

## Background

Type 1 diabetes (T1D) is a chronic autoimmune metabolic disorder resulting from the progressive destruction of insulin-producing ß-cells in the pancreatic islets of Langerhans leading to insulin deficiency, hyperglycemia, and lifelong exogenous insulin dependence. Despite appropriate glycemic management, individuals with T1D have an increased risk for severe morbidity and increased mortality compared to the general population (1, 2). There is an unmet need for effective T1D prevention and treatment strategies. Patients with T1D demonstrate histological abnormalities in the exocrine pancreas and reduced pancreas size even in pre-clinical stages of the disease (2–4). Current data suggest that in addition to the immune cell attack, other pancreatic cellular components might contribute to β-cell demise. Beta-cells interact with cells within and outside the islets of Langerhans to regulate insulin secretion and maintain glucose homeostasis (5–9). These cellular communications are essential for normal pancreatic function and for maintaining β-cell health but are affected in T1D inducing stress that could lead to neo-antigen expression and reducing β-cell resistance to the autoimmune attack (10).

Pancreatic stellate cells (PSCs) are resident mesenchymal cells of the pancreas. PSCs reside in perivascular locations, in the peri-acinar and periductal space in the exocrine pancreas, and in the endocrine pancreas. PSCs regulate extracellular matrix turnover, stimulate acinar cell secretion by producing acetylcholine in response to cholecystokinin, and produce cytokines contributing to the recruitment of inflammatory cells to the pancreas(11). Previous studies implicate PSCs in the pathobiology of major exocrine pancreatic disorders such as chronic pancreatitis and pancreatic cancer (11, 12). PSCs exist in two states, quiescent and activated. Quiescent PSCs (qPSCs) are characterized by the presence of lipid droplets rich in vitamin A. However, in case of tissue injury or insult qPSCs become activated, they lose their vitamin A-rich lipids droplets and develop a myofibroblastic phenotype. Activated PSCs (aPSCs) release factors that promote an inflammatory state and extracellular matrix deposition, leading to organ fibrosis and interfering with acinar cell function (11–13).

The plasticity of PSCs complicates *in vitro* functional studies, however single-cell RNA-sequencing (scRNA-seq) analyses can provide insights into *in situ* function. ScRNA-seq and single-nuclei RNA sequencing (snRNA-seq) analyses of pancreatic tissue enable the identification of subpopulations of cells and cellular heterogeneity within the pancreas. Given the established activation of PSC in inflammatory conditions, the increased intra-lobular and inter-acinar fibrosis in T1D, the contributions of PSCs in pancreatitis and pancreatic cancer progression (which are both associated with diabetes), we hypothesized that PSCs contribute to β-cell function decline and to the exocrine pancreas alterations characteristic of T1D. Revealing and understanding how PSC subpopulations interact with different cell types may shed light on complex regulatory networks in the pancreas. This could lead to the identification of signaling pathways implicated in T1D progression that can potentially be therapeutically targeted. Therefore, in this study we analyzed scRNA-seq FASTQ files using CellChat of 20 islet-enriched samples to compare the cell-to-cell communication in islet samples from donors with and without T1D, focusing on aPSCs. These analyses revealed changes in global communication and identified altered links in T1D involving aPSCs communication, providing evidence for a potential role of PSCs in T1D pathogenesis and exocrine pancreas alterations.

## Methods

### Human samples, collection, processing, and preparation

Data from human pancreatic islets of Langerhans scRNA-seq experiments were analyzed in this study to compare the cell communication of PSC subpopulations in T1D and controls (non-diabetes). The organ procurement, processing, islet isolation, sample preparation, and sequencing were done by the Human Pancreas Analysis Program (HPAP) as previously described (14, 15). In brief, human pancreatic islets were isolated, dissociated into a single cell suspension, barcoded, and sequenced. The samples were processed using the 10x-Chromium Single Cell 3’ Reagent Kit (10X Genomics).

The scRNA-seq raw FASTQ files were downloaded from the HPAP PANC-DB data portal from 20 donors (10 files from donors with T1D for less than 10 years and 10 files from donors without diabetes)(16). Because it is known that insulin secretion in humans is impaired with increasing age, samples were selected from donors with similar age distribution who were 30 years of age or younger (17–19). A table with key clinical characteristics and experimental samples of the HPAP donors included in this study can be found in Additional file 1 following the American Diabetes Association guidelines (20, 21). See Availability of Data section below.

#### Preliminary processing and filtering

For initial preprocessing of the FASTQ files the Cell Ranger 6.0.1 software was applied with GRCh38 as the human reference genome. Barcodes were filtered for a minimum of 500 nFeature RNA per cell and < 15% mitochondrial reads. Those cells not fulfilling these criteria were excluded from future analyses. Doublet removal was then performed using Scrublet, an R-package (22). Scrublet was ran on each sample separately as recommended to detect technical doublets formed by the random co-encapsulation of two cells. The doublet score threshold was adjusted to make sure that all predicted doublets were mostly co-localized in the same clusters on the gene expression map.

#### Ambient RNA correction

To account for extracellular RNA contamination that may get trapped in a droplet during library generation, ambient RNA removal was performed using SoupX 1.6.1 (23). SoupX was applied on the raw feature barcode matrices using the automated contamination fraction estimation method. The corrected count values for each sample were then rounded-to-integer, ensuring resulting counts remain as integers for use in downstream analyses.

#### scRNA-seq clustering and cell type annotation

Single-cell data integration and batch-effect correction were performed using Seurat 4.3.0.1 and Harmony 0.1.1 following a standardized procedure (24). Two packages were utilized in an integrated fashion where Seurat 4.3.0.1 was first applied to create Seurat objects for individual samples and then the raw Seurat objects were merged (25). Further normalization, variable feature selection, and PC calculation were performed on the merged Seurat object. With the pre-computed PCs, Harmony took the merged Seurat object as input for integration analysis using k-means clustering. Once integrated, Seurat was applied for clustering using the Leiden algorithm at 1.2 resolution. Three key steps were followed with the Seurat analysis; first, the Uniform Manifold Approximation and Projection (UMAP) was run, followed by dimensional reduction in several steps including constructing a Shared Nearest Neighbor (SNN) Graph. Finally, clusters of cells were identified by a SNN modularity optimization based on the original Louvain algorithm. Cell adjudication for all clusters was performed using recognized cell markers described in the results section.

#### Cell-to-cell communication analysis

Cell-to-cell communication analysis was performed with CellChat 2.1.2, an R package designed for inference, analysis, and visualization of cell-cell communication from single-cell data (26). The tool applies a top-down analysis approach, starting with a global portrayal of the number and strength of cellular communications, and then refining to a molecular signaling pathway (26). In this analysis, two separate CellChat objects were created with Secreted Signaling and top 15000 gene features, for each of the two sample groups in our experiment, T1D and controls. Secreted Signaling is a subset of CellChatDB, the knowledge database underlying CellChat. It accounts for ~ 40% of 3300 validated molecular interactions in the database, representing manually curated autocrine/paracrine signaling interactions. The FindVariableFeatures from the Seurat package was applied to identify the top 15000 genes. The objective was to make the analysis procedures computationally feasible while maintaining useful information with reduced random noise. The average gene expression per cell group and modeling of the probability of cell-cell communication was performed utilizing the trimean. The minimum number of cells required in each cell group for cell-cell communication analysis was set to be 10% to filter out noise cell-cell communication signaling. This CellChat analysis is based on joint manifold learning, a subfield of machine learning that operates in continuous domains and learns from observations (27). The signaling networks in T1D and control are represented as points in a Euclidean space in which a larger distance implies a larger difference in the communication networks between the two groups. In addition, we performed the analysis with functional and structural similarity, and we identified the signaling networks using rankSimilarity (R). The CellChat objectives for T1D and the control sample groups were merged for comparative analysis in cell communication, such as surveying altered interactions and cell populations, determining altered signaling with distinct network architecture and interaction strength, and identifying the up-regulated and down-regulated signaling ligand-receptor pairs by differential expressed gene analysis.

### Gene set enrichment analysis (GSEA)

Differentially expressed genes (DEG) between T1D and controls in aPSCs were identified with MAST, a hurdle model specifically tailored to scRNA-seq data (28). Gene set enrichment analysis (GSEA) was subsequently performed with ClusterProfiler 4.10.1 to identify highly enriched pathways and biological processes (29).

### Statistical analysis

Statistical significance analyses were performed using tests contained within the Seurat, CellChat, and GSEA packages.

## Results

### Clustering of human islet samples segregates populations of mesenchymal cells

**Figure 1:**
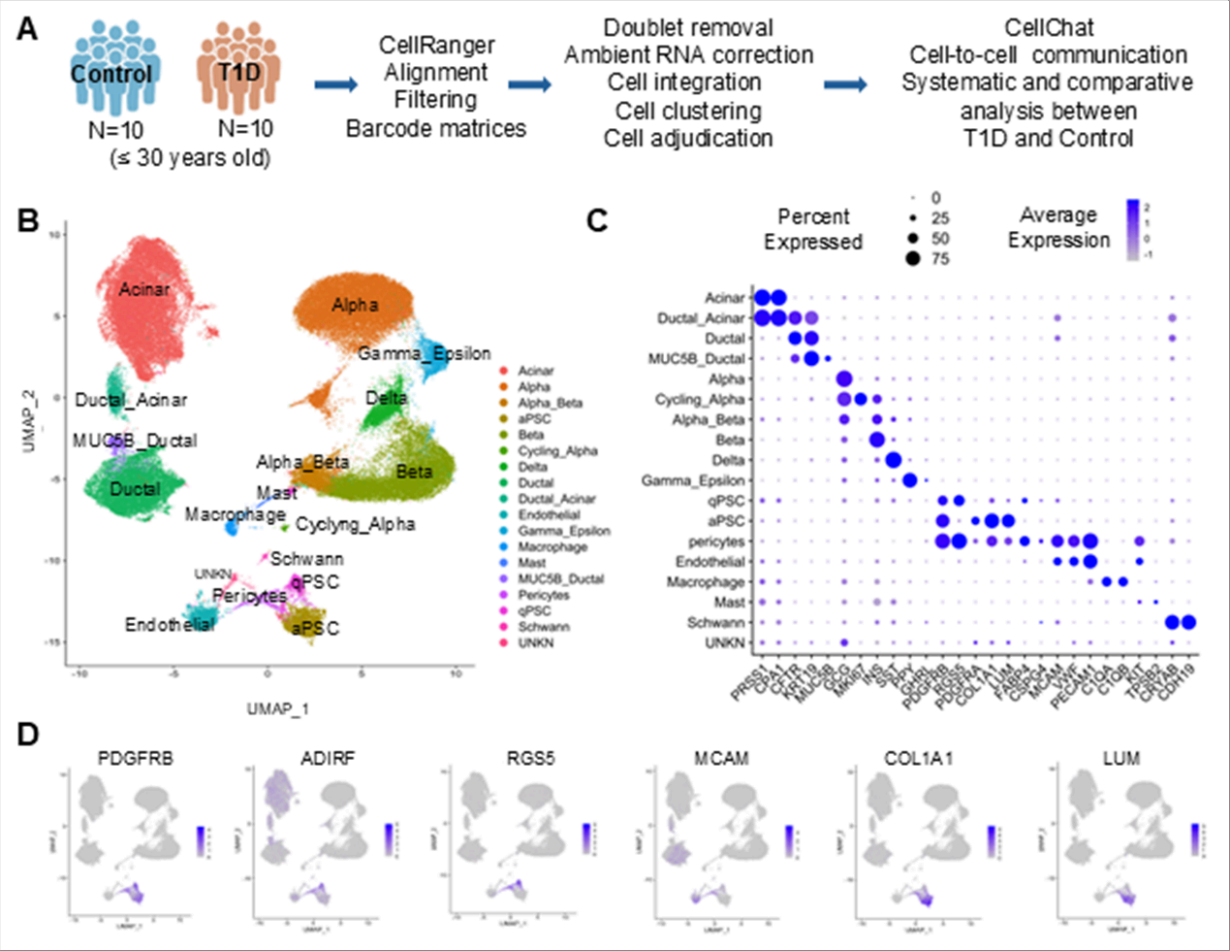
Study design, cell clustering, and cell adjudication. A) Diagram of the analyses performed with the scRNA-seq data of pancreatic islet from T1DM and controls organ donors from HPAP. B) Uniform manifold approximation and projection (UMAP) visualization of clustering in gene expression space with cell adjudication. C) Dot plot of normalized average expression and expression percentage of cells for the marker genes in each cluster. D) Dimensional reduction plots for representative genes in the islet enriched scRNA-seq data of the three mesenchymal clusters. Platelet derived growth factor receptor beta (PDGFRB), ADIRF, RG5, MCAM, LUM, COL1A1.

We analyzed scRNA-seq data from 10 T1D and 10 control donors and obtained a UMAP with 100345 cells and 18 clusters that we adjudicated using conventional cell markers including α (GCG), β (INS), δ (SST), γ/ε (PPY, GHRL), α/β (GCG/INS), cycling α (GCG, MK167), endothelial (VWF), macrophage (C1QA/B), mast (KIT, TSBP2), acinar (PRSS1, CPA1), ductal (CFTR, KRT19), MUC5B ductal (CFTR, MUC5B), ductal/acinar (CFTR, KRT19, PRSS1, CPA1), and Schwann (S100B, PLP1) cells. (Fig. 1A–C). Mesenchymal clusters were identified using expression levels of cell-specific biomarkers described in the literature. Along with PDGFRB, qPSCs expressed RGS5, ADIRF and FABP4 while aPSCs expressed PDGFRA, LUM, COL1A1, and pericytes expressed CSPG4, MCAM and RGS5 (Fig. 1C,D) (11, 30–33). We could not adjudicate one cluster, so it was labeled unknown. In addition, we retrieved the top differentially expressed genes in each mesenchymal population compared to the rest of the cells which we provide as tables in the Additional File 2.

### Increased cell-cell interaction dynamics in T1D compared to controls

**Figure 2:**
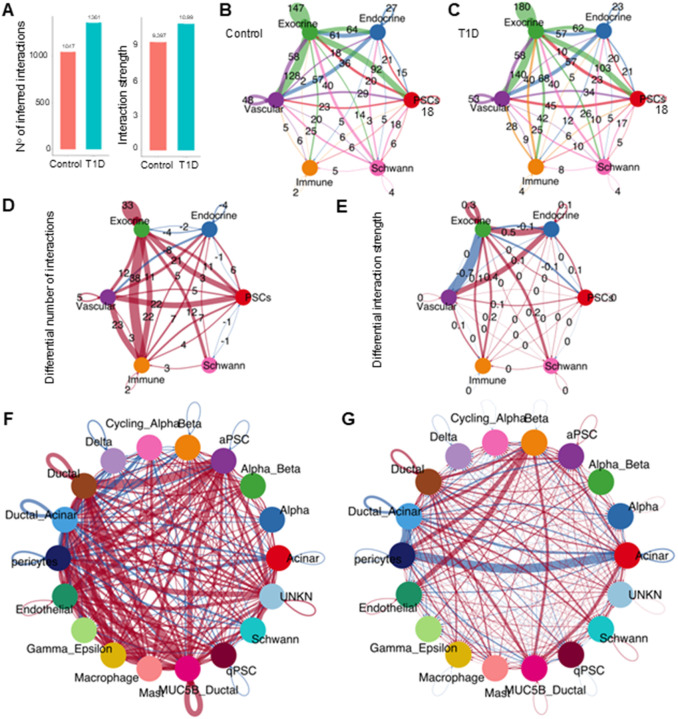
Global pancreatic cell-cell interactions. A) Bar plots for the total number of interactions and interaction strength of the inferred cell-cell communication networks in T1D compared to control set. B) Simplified circle plot for the total number of interactions of defined cell groups in the control samples. The color of the lines retains the color assigned source cell group. C) Simplified circle plot for the total number of interactions of defined cell groups in the T1D samples. The color of the lines retains the assigned cell group/dot color. D) Simplified circle plots for the total number of differential interactions in the cell-group communication network. E) Simplified circle plots for the differential interaction strength in the cell-group communication network. F) Circle plots for the differential number of interactions for the individual cell-cell communication in T1D compared to the control set. G) Circle plots for the differential interaction strength for the individual cell-cell communication in T1D compared to the control set. For the differential plots (D, E, F, G) red lines represent increased signaling and blue lines represent decreased signaling in T1D compared to control set. The thickness of the lines represents the communication probability.

We applied CellChat to identify altered cellular communications in T1D. Overall, the T1D set demonstrated a greater number of interactions (1361 vs 1047) and interaction strength (10.99 vs 9.40) compared to controls (Fig. 2A). These differences were more apparent when the adjudicated cell types were aggregated into 6 broad functional categories: endocrine, exocrine, PSCs, vascular, immune, and Schwann cells (representing neural function) (Table 1).

In both control and T1D sets, the exocrine compartment showed the largest number of interactions (Table 2 and Fig. 2B, D).

Compared to the control set, the T1D set showed a global increase in the number of interactions among the exocrine, immune, vascular, and PSCs groups. Focusing on the endocrine group, there were fewer interactions with the exocrine group, the vascular cells, PSCs, and among the endocrine cellular components themselves (Fig. 2D). Interestingly, the larger increase in the number of communications involving the immune group in T1D was with the exocrine group, totaling an increase of 60 interactions in T1D compared to control (Fig. 2D). Analyzing the differential strength of the interactions, we found an increased strength in the interactions between the endocrine group with the vascular and exocrine groups, whereas the larger decrease in strength was between the exocrine and vascular groups (Fig. 2E).

The differential number of cell-to-cell interactions of the individual cell types between control and T1D data sets demonstrated the complexity of cellular communication within the pancreas (Fig. 2F). Activated PSCs play a key role in T1D with a major increase in cell-to-cell interactions with ductal cells, MUC5B ductal, pericytes and endothelial cells which are further investigated in the following sections (Fig. 2F). Multiple human scRNA-seq and snRNA-seq studies reported that the most significant changes in gene expression in samples from T1D donors occur in β-cells (32, 34–36). As expected then, β-cells showed the largest interaction strength increase in the T1D compared to the control set, in interactions with endothelial cells, pericytes, ductal-acinar cells, and MUC5B-ductal cells. The major decrease in signaling strength in T1D was between acinar cells and pericytes (Fig. 2G). Closer examination of the differential number of interactions among different cell populations between the T1D and control sets showed that the MUC5B-ductal cells and aPSCs sent the largest differential outgoing signal, whereas the cells that received the largest differential signal in T1D compared to control were the ductal, ductal-acinar cells, pericytes, endothelial and aPSCs (Additional file 3). Overall, this data shows new signals outgoing from the PSCs and altered/decreased interactions between the acinar cells and pericytes in T1D. At the same time, the β-cells maintain a central role sending signals with the highest strength particularly to endothelial cells and pericytes already described being altered in T1D (37).

#### Cell signaling in T1D shows a predominance of cytokine pathways from aPSCs.

**Figure 3:**
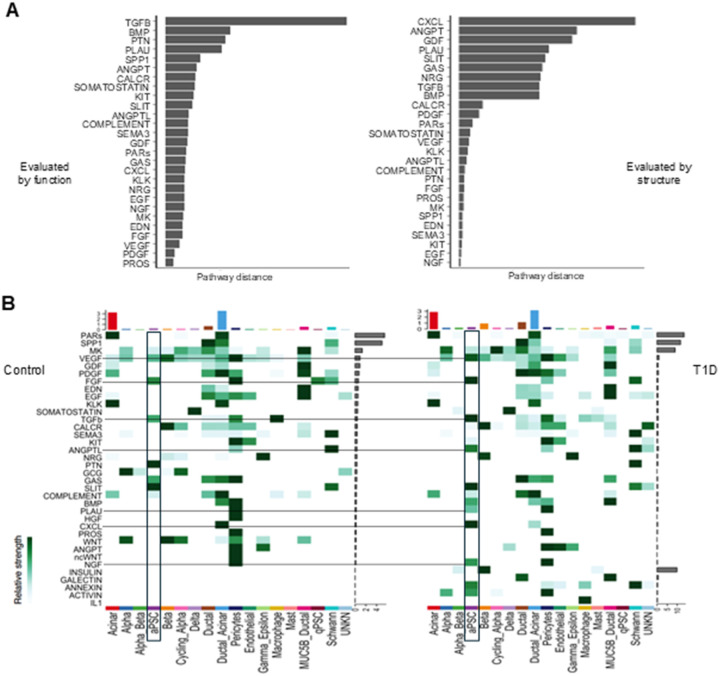
Differential interactions and pathways in T1D compared to control samples. A) Barplots for the ranking of difference between T1D and control in the shared two-dimensions space, evaluated by function (Left) and structure (Right) based on their Euclidean distance mined from joint manifold learning. B) Heatmap of the patterns of outgoing signaling in controls and T1D (values are row scaled). The colored bar plot at the top shows the total signaling strength of the cell group summarizing all the signaling pathways displayed in the heatmap. The grey bar plotted at the right of the control and T1D maps shows the total signaling strength of the signaling pathway by summarizing all cell groups displayed in the heatmap. The aPSC are demarcated by a black box.

Subsequently, we performed comparative analyses of the signaling networks significantly altered in T1D. The top signaling networks by functional assessment were TGFB, BMP, PTN, PLAU, SPP1, and ANGPT, and by structural assessment were CXCL, ANGPT, GDF, PLAU, SLIT, and GAS (Fig. 3A). Interestingly, ANGPT and PLAU were among the top signaling pathways underlying the disease status evaluated by both functional and structural approaches. Analysis of the pattern of outgoing molecular signaling showed that the acinar and ductal-acinar cells presented the largest total signal strength in control and T1D states, with the PARs signaling dominant (Fig. 3B). As expected for the T1D data, set due to the active inflammatory and autoimmune activity, the interleukin 1 (IL1) pathway from macrophages was increased. Examining the mesenchymal cells, aPSCs outgoing signaling patterns were increased in number and strength in T1D (Fig. 3B). The major increase in signaling strength in aPSCs in T1D was found in the CXCL, ANGPTL, and TGFB pathways. Conversely, qPSCs greatly reduced fibroblast growth factor (FGF) signaling strength in T1D (Fig. 3B). The most active signaling pathways in T1D, including those from aPSCs, are consistent with inflammatory activation.

### Upregulated signaling pathways support an active role of aPSC in T1D

To explore how different pancreatic cells’ signaling pathways are functionally organized, we set to identify communication patterns and key signals with CellChat pattern recognition. We established 5 patterns of communication in the control set (k = 5).

**Figure 4:**
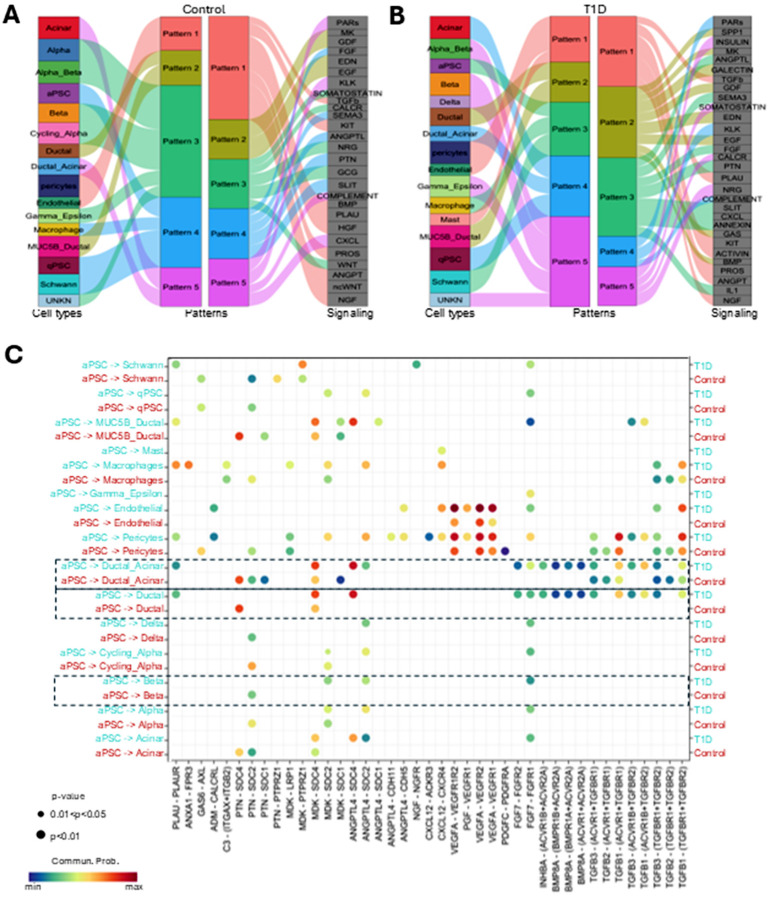
Increased signaling from aPSCs in T1D. A) Alluvian plot of the inferred outgoing communication pattern of secreting cells in the control set showing the correspondence among patterns and cell types. The thickness of the flow represents the contribution of the cell type or signaling pathway. B) Alluvian plot for the inferred outgoing communication pattern of secreting cells in the T1D set. C) Bubble plot of upregulated ligand-receptor signaling based on a differential expression analysis, considering the aPSCs as senders, ligand source.

The aPSCs shared signaling patterns with macrophages and Schwann cells in both controls and T1D characterized by pathways like SLIT, pleiotrophin (PTN), semaphorin 3 (SEMA3), and FGF (Fig. 4AB). In controls aPSCs also shared a communication pattern with qPSCs but not in T1D (Fig. 4A). The qPSCs in T1D aligned with acinar and ductal-acinar cells with strong PARs, KLK and complement signaling. The aPSCs in T1D shared a communication pattern with macrophages characterized by ANGPTL, SEMA3, PTN, CXCL and IL1, indicative of a pro-inflammatory phenotype. Interestingly, in T1D, mast cells signal with the same communication pattern of pericytes and endothelial cells, characterized by the PLAU, KIT, ACTIVIN and ANGPT signaling (Fig. 4B).

Because the aPSCs showed the largest number of differential interactions in T1D compared to the control data set, we further investigated their outgoing molecular signals and the recipient cells by analyzing ligand-receptor pairs. Based on differential expression analysis, we found the most upregulated signaling in the T1D set from aPSCs were with ductal, ductal-acinar, pericytes, and endothelial cells. Notably, MDK and angiopoietin-like protein 4 (ANGPTL4) from aPSCs interacted with SDC4 receptors in acinar, ductal, ductal-acinar, and MUC5B-ductal cells (Fig. 4C). Some interactions were unique to T1D like those of MDK and ANGPTL4 from aPSCs with SDC2 receptors in β-cells (Fig. 4C). The most significant difference in cell communication probability was via the ligand-receptor pair ANGPTL4-SDC4, which was missing in the control data set. TGFB signaling from aPSCs to the ductal cell populations was also significantly upregulated in T1D. In contrast, PTN-SDC4 ligand-receptor communication with the ductal cell clusters was absent in T1D and upregulated in controls. The PTN-SDC2 ligand-receptor pair interaction displayed a similar trend but with a broader recipient cell spectrum (endocrine cells, qPSCs, Schwann cells). VEGFA signaling from aPSCs demonstrated a high communication probability with the VEGFR1 and VEGFR2 of pericytes and endothelial cells in controls and even greater in T1D (Fig. 4C). Interestingly, PLAU-PLAUR communication between aPSCs and macrophages was only upregulated in the T1D set.

The analysis of communication from aPSCs to β-cells showed differential cell communication between T1DM and control (Table 3). We found three ligand-receptor pairs only present in T1D with moderate probability: ANGPTL4-SDC2, MDK-SDC2 and FGF7-FGFR1, whereas we found the PTN-SDC2 interaction was only present in the control set (Table 3 and Fig. 4C). The inference of MDK-SDC2 and ANGPTL4-SDC2, interactions from aPSCs to β-cells which was only present in the T1D set suggests a larger contribution of aPSCs to T1D progression than previously recognized with a dual role, directly acting on the β-cells and indirectly sustaining chronic inflammation (Table 3).

Additional analysis of the upregulated ligand-receptor pairs from aPSCs with the 17 other cell types showed 13 ligands forming 79 ligand-receptor pairs upregulated in T1D. ANGPTL4 was the upregulated ligand with the largest spectrum, interacting with multiple receptors in 9 cell types. In contrast, nerve growth factor (NGF) secreted by aPSCs only interacted with the NGFR on the Schwann cells (Fig. 4C, and Additional file 4 A). We found 6 ligands forming 46 ligand-receptor pairs downregulated in T1D compared to control samples. Two of the ligands, PTN and FGF2 from aPSCs, were broad-spectrum-ligands downregulated in T1D (Fig. 4C and Additional file 4 B). Overall, we found more signaling pathways upregulated than downregulated in the T1D set compared to the control set, and aPSCs were major contributors, supporting our hypothesis of an active role of aPSCs in the pro-inflammatory T1D environment.

#### Secreted factors involved in lipid metabolism, inflammation, glucose homeostasis and fibrosis from aPSCs are upregulated in T1D

**Figure 5:**
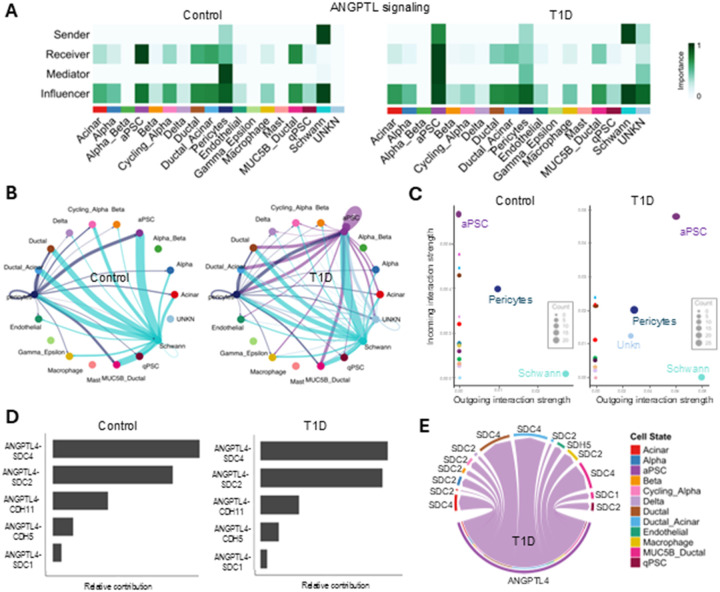
aPSCs and Schwann cells upregulate Angiopoietin-Like protein signaling in T1D. A) Heatmap of the network centrality analysis for the ANGPTL pathway in control and T1D samples. B) Circle plots of the statistically significant intercellular signaling interactions for the ANGPTL pathway in control and T1D samples. The color of each circle/dot represents one cell type; Lines (edges) connecting circles represent significant intercellular signaling inferred between those cell types. The color of the line corelates the sending cell color. C) Scatter plot of the cell populations sending or receiving ANGPTL signals in the control and T1D sets. D) Bar graph of the contribution of each ligand-receptor pain in control and T1D samples. E) Chord diagram of the ANGPTL signaling ligand-receptor pairs in T1D based on the differential expression analysis.

We further analyzed the ANGPTL signaling network that was among the top upregulated signaling pathways from aPSCs (Fig. 4B). ANGPTL is involved in lipid metabolism, inflammation, glucose homeostasis and fibrosis. In the control set, Schwann cells were identified by network centrality measures as major secretors of ANGPTL, followed by pericytes. At the same time aPSCs received significantly more signaling than endothelial cells (Fig. 5A). In contrast, in T1D the aPSCs are key senders, receivers, mediators, and influencers of the ANGPTL signaling network displaying a broader activity in T1D (Fig. 5A). Examination of the ANGPTL cell-cell communication in circle plots demonstrated the upregulation of ANGPTL pathway in T1D with autocrine and paracrine signaling from aPSCs and Schwann cells. Importantly, it also confirmed the newly identified direct communication from aPSCs to β-cells in T1D (Fig. 5B). The significant changes in sending or receiving ANGPTL signals between the control and T1D sets emphasized a key role of Schwann cells in the function of normal tissue as a ligand source. This dominance was shared with the aPSCs which became strong senders and receivers in T1D (Fig. 5C). In the control set, the ANGPTL4-SDC4 ligand-receptor pair was the main interaction, whereas in T1D, the contribution of ANGPTL4-SDC2 ligand-receptor pair increased and was equivalent to the ANGPTL4-SDC4 contribution (Fig. 5D). The aPSCs autocrine loop and the interaction with the β-cells were mediated by ANGPTL4-SDC2 receptor, while the interaction with Ductal and Ductal-Acinar cells was mediated by SDC4 receptors (Fig. 5E). These findings revealed the direct interaction of aPSCs with β-cells via ANGPTL4-SDC2, suggesting a potential role of the angiopoietin-like proteins activity in β-cell dysfunction.

**Figure 6:**
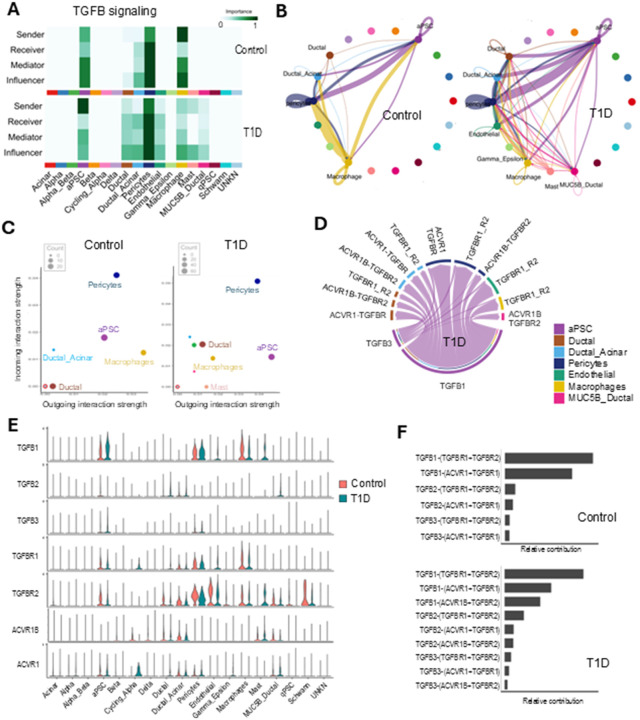
TGFB signaling is upregulated in T1D. A) Heatmap of the network centrality analysis for the TGFB pathway in the control and T1D sets. B) Circle plots of the statistically significant intercellular TGFB signaling interactions in control and T1D. The color of each circle/dot represents one cell type. Lines (edges) connecting the dots represent significant intercellular signaling inferred between those cell types. The color of the line corelates with the color of the sending cells. C) Scatter plot of the cell populations sending or receiving TGFB signals in the lcontrol and T1D sets. D) Chord diagram of the up-regulated TGFB signaling ligand-receptor pairs in T1D based on the differential expression analysis. E) Violin plot of TGFB signaling gene expression distribution among the cell types. F) Bar graph of the contribution of each ligand-receptor pair in control and T1D samples.

Transforming growth factor β (TGFβ) is known to activate qPSCs and elicit profibrotic signaling in the pancreas (38). Since TGFB signaling emerged as the top signaling pathway in the T1D set functional analysis, we further analyzed it. The heatmap of the relative importance of each cell group based on the computed sender, receiver, mediator and influencer measures of the TGFB signaling network revealed macrophages as the main initiators of the pathway followed by pericytes and aPSCs in the control set. In contrast, in T1D, aPSCs secreted TGFB with the highest strength and more cell types were affected by it as expected in a pro-inflammatory environment (Fig. 6A). In the control and T1D sets, aPSCs secreted TGFB and displayed an autocrine activation loop and paracrine network. In T1D the TGFB signaling was upregulated, mainly between aPSCs and pericytes and with lesser strength with ductal, ductal-acinar, endothelial, MUC5B-ductal cells, and macrophages (Fig. 6B). In T1D aPSCs TGFB1 was upregulated compared to control (log2FC: 0.69 with adjusted p.Val: 1.76*10^−56). Pericytes showed strong autocrine signaling in the control set which remained unchanged in T1D. While outgoing TGFB signaling from macrophages in the control samples was strong, it decreased in the T1D set. Conversely, aPSCs became the cell type with the greatest outgoing TGFB signaling in T1D (Fig. 6B, C). Analysis of the ligand-receptor pairs in T1D revealed that TGFB1 and TGFB3 ligands interacted with TGFBR1, TGFBR2, ACVR1, and ACVR1B in multiple cell types (Fig. 6D) with predominant expression of TGFB1. The main receptor for the TGFB ligands in the pancreas was TGFBR2 (Fig. 6E, F). Although the ACVR1B receptor, also known as activin receptor-like kinase 4 (ALK4), was expressed in the control and T1D sets, only in the T1D set it was predicted to interact with the TGFB ligands (Fig. 6F).

#### CXCL and NGF associated with immune cell recruitment, cell growth, and differentiation are secreted by aPSC particularly in T1D

We identified 960 differentially significant ligand receptor pair interactions among the 18 cell types in the human pancreatic islet samples (with ligand p.Val of 0.01 and ligand logFC of 0.05) and specifically, 166 for aPSCs as a ligand source. Only two ligands sent by aPSCs in the T1D set were absent in the control set, CXCL and NGF.

**Figure 7:**
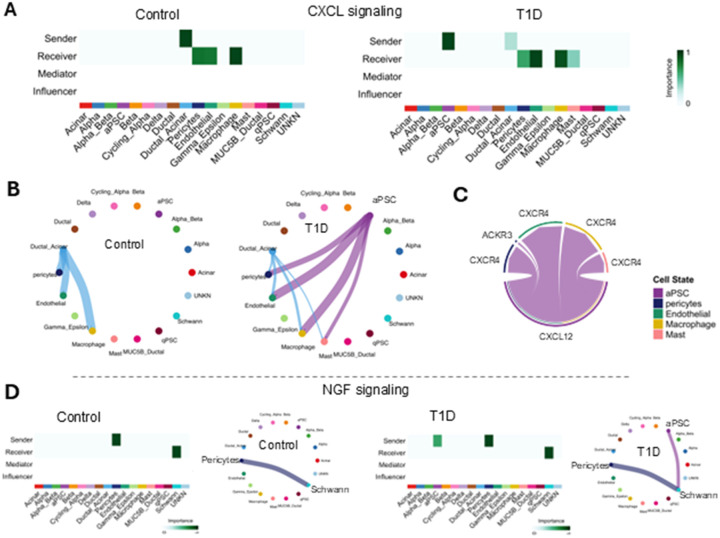
Upregulation of CXCL and NGF signaling from aPSCs in T1D. A) Heatmap for the CXCL signaling network centrality analysis for each cell type in the control set and T1D set. B) Circle plots of the statistically significant intercellular CXCL signaling in the control and T1D sets. The color of each dot represents a cell type. Lines connecting the circles represent the signaling inferred between those cell types. The color of the line corelates with the color of the sending cells. C) Chord diagram of the up-regulated CXCL signaling ligand-receptor pairs in T1D based on the differential expression analysis. D) Heatmap for the NGF signaling network centrality analysis for each cell type and corresponding circle plot in the control set and the T1D set.

In controls, CXCL signaling was mainly driven by ductal-acinar cells, which sent signals to pericytes, endothelial cells, and macrophages. In contrast, in T1D, aPSCs became the dominant source of CXCL signaling, sending stronger signals than ductal-acinar cells to pericytes, endothelial cells, macrophages, and mast cells (Fig. 7A, B). The CXCL signaling pathway plays a crucial role in inflammation and immune responses. Interestingly, in these samples, no mediators or influencer cells were identified for the CXCL network which would suggest a spatially close sender-receiver interaction. The main CXCL ligand secreted by the aPSCs and ductal-acinar cells was CXCL12, which interacted with the CXCR4 receptor in pericytes, endothelial cells, macrophages, and mast cells and the ACKR3 receptors in pericytes (Fig. 7C). Accordingly, the CXCL signaling inference suggests that the local CXCL12-CXCR4 axis upregulated in the aPSCs in T1D might assist in the recruitment of immune cells to the pancreas, further contributing to the characteristic T1D inflammatory milieu.

The other signaling pathway in which aPSCs were secretors only in T1D was the NGF pathway. In the control set, pericytes sent NGF that was received by Schwann cells, whereas in the T1D set the aPSCs contributed to the NGF signaling interacting with the NGFR of the Schwann cells (Fig. 7D). Given the NGF role of growth regulation and differentiation of sympathetic and some sensory neurons, these results suggest neural involvement may be underappreciated in T1D progression.

### Pleiotrophin signaling switch in T1D

**Figure 8:**
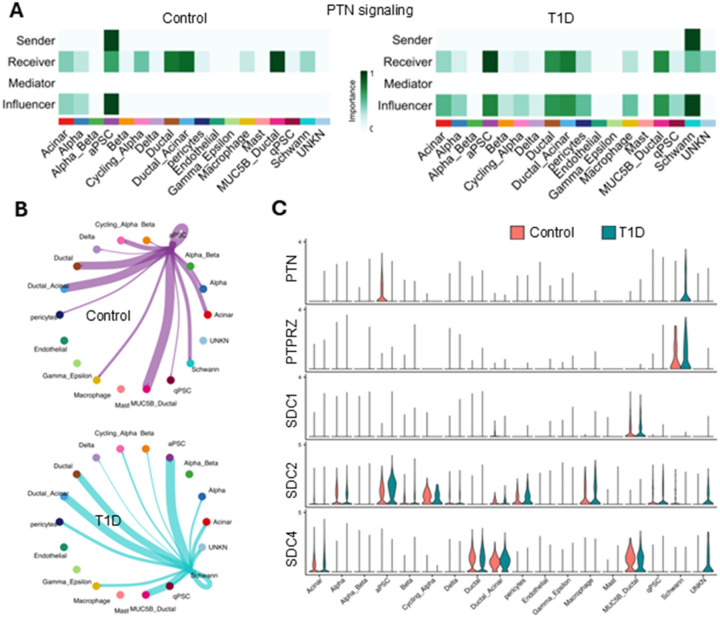
Pleiotrophin signaling switch in T1D. A) Heatmap of the PTN signaling network centrality analysis for each cell type in the control and T1D sets. B) Circle plots of the statistically significant intercellular PTN signaling interactions in the control and T1D sets. The color of each dot represents the cell type, and the lines connecting the dots represent the signaling inferred between those cell types. The color of the line corelates with the color of the sending cells. C) Volcano plot of the PTN signaling gene expression distribution between control and T1D sets.

The patterns of outgoing signaling from the aPSCs in the control set showed very strong PTN signaling. However, the outgoing PTN signaling from aPSCs was absent in the T1D set (Fig. 3B). Further analysis showed that in the control set, in the islet neighborhood aPSCs were the only cell type secreting PTN, which was received by most of the cell types, in agreement with its aforementioned broad-spectrum ligand category. This supports aPSC role in providing tissue homeostasis, cellular growth and survival. Conversely, in the T1D set, pleiotrophin signal was only sent by Schwann cells (aPSCs ceased to secrete PTN) and the signal was received and influenced by most of the pancreatic cells (Fig. 8A, B). In the control and T1D data sets, the aPSCs and Schwann cells respectively displayed autocrine PTN signaling. Once again, we found aPSCs and Schwann cells playing a key differential role in the T1D setting. The main PTN receptor in aPSCs was SDC2, whereas the major PTN receptor in Schwann cells was PTPRZ1 regardless of the sample set (Fig. 8C). SDC2 and SDC4 were the main receptors for PTN in other cell types. MUC5B-ductal cells also utilized the SDC1 receptor for pleiotrophin. All in all, the strong down regulation of PTN signaling from aPSCs suggests that pleiotrophin could become an early therapeutic target.

### Gene set enrichment analysis supports CellChat inferred aPSCs signaling upregulation in T1D compared to ND

**Figure 9:**
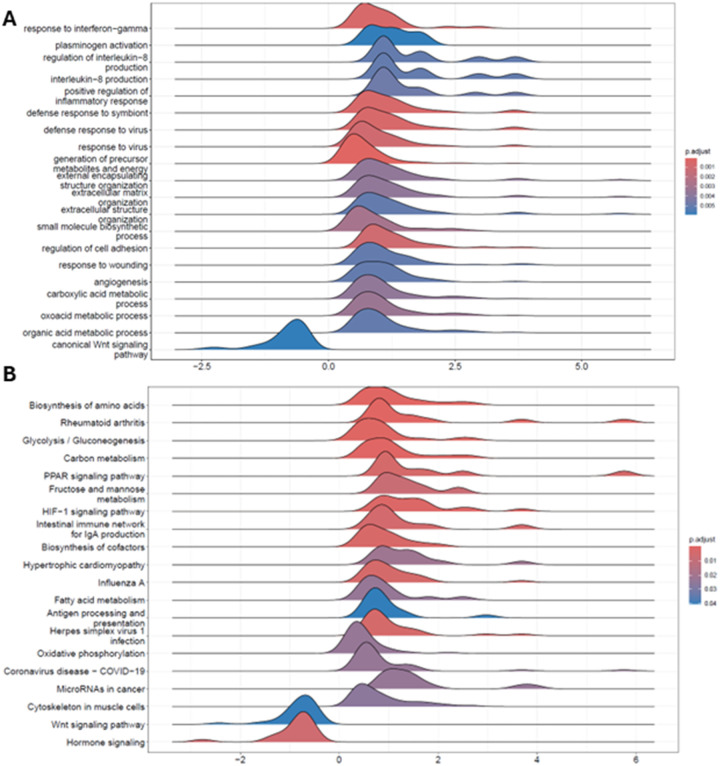
Gene set enrichment analysis of aPSCs support CellChat cellular signaling upregulation in T1D compared to ND. A) Ridge plot for the distribution of GSEA of the Gene Ontology biological process collection comparing the scRNA-seq data for the aPSCs cluster from donors with and without T1D. B) Ridge plot of the enrichment analysis performed with KEGG to categorize the genes into biological pathways.

To evaluate the CellChat inferred interactions we performed DEG and GSEA on the gene expression of the aPSCs cluster identified from the scRNA-seq data. The differential gene expression analysis aligned with the significant global upregulation of biological processes (Fig. 9A, B). The Gene Ontology (GO) Biological Process differential analysis showed an enrichment for Response to interferon gamma and Positive regulation of inflammatory response. These findings align with known pathogenetic mechanisms of T1D (39–43). Additionally, processes related to extracellular matrix organization and structural remodeling were significantly enriched, underscoring the key role of aPSCs in T1D in the altered extracellular matrix described in T1D (44, 45). Several processes indicated angiogenesis in line with changes of pericytes and endothelial cells interactions (Fig. 9A).

The GSEA with the Kyoto Encyclopedia of Genes and Genomes (KEGG) collections showed many activated metabolic processes such as biosynthesis of amino acids, fructose and mannose metabolism, PPAR signaling and fatty acid metabolism that align with ANGPTL4 functions (Fig. 9B). GSEA analyses revealed downregulation of the WNT signaling pathway in T1D, supporting CellChat’s prediction of reduced PTN secretion by aPSCs in T1D (Fig. 8 and Fig. 9A, B). Since PTN and WNT pathways are interconnected, this reduction may impact WNT activity. The WNT ligand signals through the frizzled receptors to inhibit glycogen synthase kinase 3β (GSK-3β), preventing β-catenin degradation allowing its translocation to the nucleus and promoting gene expression (46). Yet, PTN can activate GSK-3β, leading to β-catenin phosphorylation, ubiquitination, and degradation and thus inactivating the WNT signaling pathway (47). Additionally, several enriched processes in both the GO.BP and KEGG analyses were associated with CellChat inferred upregulation of ANGPTL4 signaling in T1D, including processes related to response to hypoxia and PPAR signaling, known to stimulate ANGPTL4 expression (see Additional file 5).

## Discussion

Growing evidence suggests that the pancreatic microenvironment of β-cells plays a key role in their function, and every cell in the pancreas either contributes to or is affected by this microenvironment (48). Therefore, in this work, we studied single-cell transcriptomic data from donors with and without T1D analyzing their cell-to-cell communication with a focus on aPSCs. Considering their ability to release cytokines, interact with myeloid cells to promote inflammation, and alter the extracellular matrix in pancreatic diseases, we hypothesized that aPSCs might also contribute to the pathogenesis or progression of T1D. Overall our analysis found known cell-to-cell communication pathways in T1D and revealed new key cellular and molecular contributors.

Consistent with previous scRNA transcriptomic studies using islet samples, we identified the aPSCs and qPSCs clusters (31, 32, 35). Moreover, we identified the pericyte cluster as the only cluster with CSPG4 expression, also known as neuron-glial antigen 2 (NG2), a widely recognized pericyte marker. The pericytes, sometimes called mural cells, might have been missed in other pancreatic islet adjudication experiments because they segregate into the other mesenchymal clusters given their plasticity and the fact that they are a very heterogeneous cell population (49–52).

In T1D, aPSCs showed the greatest number of upregulated cell-to-cell communications, mainly with ductal, pericyte, and endothelial cells. Our analysis uncovered a large increase in the number of interactions, largely from the exocrine compartment, among all cells in T1D compared to controls (Fig. 2B–D). Recently, using snRNA-seq data Melton et al. found a reduction in the number of interactions in recent-onset and long-standing T1D compared to control, mainly driven by exocrine cells (10). We believe this discrepancy is due to the different types of samples analyzed. In a healthy pancreas, the exocrine cells make up approximately 80–98% of all cells, whereas in T1D there is a marked decrease in acinar cell number with an increase of fibrosis (53). In our study we analyzed islet-enriched samples, leveling the exocrine cell and islet number contributions in both groups and highlighting differential interactions. This is supported by the fact that the endocrine cells displayed significant increases in both outgoing and incoming signaling in recent-onset T1D (10).

It is widely known that the cytokine milieu is enhanced in the pancreas in T1D and multiple publications have analyzed circulating cytokine levels in humans with T1D (5, 54–57). Consistent with this, we found TGFB cytokine signaling significantly upregulated with enhanced cell communication in T1D compared to controls (Fig. 3A, B, 4C, 6). Furthermore, TGFB was the pathway with the highest number of ligand-receptor pairs detected in T1D. In samples from control donors, TGFB ligands were secreted by macrophages, pericytes and aPSCs (in order of signal importance). However, in samples from donors with T1D, aPSCs were the major secretors (Fig. 3B, 4C, 6). TGFβ is usually considered an anti-inflammatory cytokine that contributes to immune tolerance, suppressing autoreactive T cells and promoting the development of regulatory T cells which could be therapeutically exploited for delaying T1D development (58). In this fashion aPSC could be acting as mesenchymal supporters of pancreatic tissue health. However, higher levels of TFGβ in the islets lead to severe fibrosis, β-cell failure, and increase β-cell susceptibility to apoptosis (59–61). Although TGFB plays a complex role in T1D, its upregulation in diabetic conditions aligns with existing literature. In humans, elevated circulating TGFβ1 levels and increased expression of TGFβ1-regulated miRNAs was observed in patients with T1D compared to individuals without diabetes, and these changes were associated with accelerated progression of diabetic nephropathy (62–65). Additionally, the increase of advanced glycation end products (AGEs) that occurs in diabetes, interact with their receptor for advanced glycation end products (RAGE), which in turn increases TGFβ expression in aPSCs and other cells (66, 67). It is well established that TGFβ activates PSCs. Therefore, in the context of T1D, the elevated levels of TGFβ suggest a sustain activation of aPSCs through both paracrine and autocrine signaling, which in turn promotes continuous and increased secretion of TGFβ by aPSCs (68, 69). While the aberrant TGFB expression in aPSCs may contribute to the loss of tolerance in T1D, we cannot exclude that the expression is secondary to T1D hyperglycemia and may contribute to chronic diabetic complications. Apart from this, the upregulation of TGFβ in aPSCs, causes autocrine upregulation of extracellular matrix production and inhibition of collagen degradation, establishing a positive loop of fibrosis and inflammatory signaling in the T1D pancreas (68, 70).

CellChat inferred the ANGPTL signaling network as being among the top pathways by functional and structural assessment (Fig. 3A) and among the major aPSCs outgoing signaling increases in T1D (Fig. 3B). The ANGPTL gene family encode angiopoietin-like proteins with important roles in lipid and glucose metabolism, insulin sensitivity, inflammation, and angiogenesis, proliferation inhibition, migration, tubule formation of endothelial cells, all of which play relevant roles in T1D pathogenesis and progression (71–73). Notably, analysis of the upregulated ligand-receptor signaling based on a differential expression analysis considering the aPSCs as ligand source, identified ANGPTL4 as the ligand interacting with syndecans and cadherins on endocrine cells (including β-cells), macrophages, and pericytes only in T1D, and with strong probability with the various clusters of ductal cells (Fig. 4C). These interactions resonate with ANGPTL4 functions. ANGPTL4 is expressed during fasting in the liver and adipose tissue via peroxisome proliferator-activated receptor (PPAR) regulation and through hypoxia-inducible factor-1α (HIF-1α) during hypoxia (74–76). In the pancreas, hypoxia can develop because of inflammation, vascular dysfunction, hyperglycemia, and immune cell infiltration. Furthermore, hypoxia plays a significant role in the development and progression of T1D, contributing to the pathogenesis of the disease by aggravating β-cell dysfunction and autoimmune response activation (77–79). Because of the role of ANGPTL4 in lipid metabolism, ANGPTL4 signaling was widely studied in the context of obesity, cardiovascular disease, and type 2 diabetes (76). However, due to contradictory findings from a few human and mouse studies in T1D, our understanding of ANGPTL4 signaling in T1D remains limited. Studies of the C-terminus ANGPTL4 showed a complex and dual role in inflammation, and its exact mechanism is not fully understood. When the pancreas is inflamed, as in acute pancreatitis, ANGPTL4 aggravates organ inflammation by inducing acinar cell damage and releasing large amounts of inflammatory cytokines. Patients with pancreatitis have elevated ANGPTL4 in the circulation and in pancreatic tissues (80, 81). Diabetes is also considered a chronic low-grade inflammatory disease characterized by increased levels of cytokines. Interestingly, in humans, variants that inactivate ANGPTL4 were associated with improved glucose homeostasis and a decreased risk of diabetes (82). ANGPTL4 inhibitors in the pharmaceutical pipeline, could mitigate inflammation in T1D pathogenesis (83). Ultimately, aggregating the upregulation of ANGPTL and TGFB signaling pathways from aPSCs with ductal and ductal-acinar cells, is in agreement with known exocrine dysregulation in pre-symptomatic and symptomatic T1D (84). Moreover, the aPSCs alterations in T1D could explain the histological and clinical dysfunction of the exocrine pancreas (3, 85, 86). It should be mentioned that pericytes also secreted many cytokines although at diverse strength, and CellChat predictions of differential cell-to-cell interactions in T1D reflected their demonstrated role on endothelial cells, altered islet blood flow, and β-cell maturation (37, 87–89).

The interesting inference of MDK-SDC2 interaction from aPSCs to β-cells and pericytes only in the T1D data set and upregulated in T1D between the aPSCs and macrophages deserves further investigation (Fig. 4C and Table 3). MDK also characterized the outgoing communication pattern of ductal and MUC5B-ductal cells (Fig. 4A, B). The MDK gene encodes the MK protein, also known as midkine, which is a pleiotropic cytokine involved in cell growth, migration, cell differentiation, angiogenesis and regulation of inflammatory response (90, 91). Based on midkine participation in chronic inflammatory diseases such as diabetic nephropathy, rheumatoid arthritis, and Crohn’s disease, midkine could facilitate the recruitment of neutrophils and macrophages to the pancreas, which could be relevant for the T1D inflammatory pancreatic environment (92–95). Moreover, based on midkine involvement in the onset and progression of autoimmune diseases like lupus erythematosus, Sjögren’s syndrome, and multiple sclerosis, midkine could contribute to inhibiting the differentiation of regulatory T cells in T1D (96, 97).

Lastly, CellChat inferred a novel and understudied interaction in the pancreas - the NGF secreted by aPSCs only in samples from individuals with T1D interacting with the NGFR, also known as p75^NTR^, on Schwann cells (Fig. 7D). Interestingly, in agreement with TGFB upregulation in T1D and highlighting the importance of pathways crosstalk, TGF-β was shown to induce NGF expression in an immortalized human PSC line and primary rat PSCs (98). The observation that pericytes secreted NGF that interacted with Schwann cells in samples from control donors, while in T1D donor samples aPSCs also secreted NGF, suggests that aPSCs might play distinct roles depending on the context. One possibility is that aPSCs work as supportive cells in the pathological state expressing NGF to counteract inflammation and promote Schwann cell survival and neuronal nurturing. Alternative aPSCs could act as a foe, mediating Schwann cell death and promoting neuronal dysfunction. Given that it is known that autonomic afferent and efferent signals play important roles in regulating systemic metabolism and homeostasis and are altered in T1D, the role of aPSCs secreted NGF in T1D requires further investigation (99–101). The islets of Langerhans are mainly innervated by sympathetic axons, and individuals with T1D and recent onset T1D demonstrate a reduced number of sympathetic axons. In contrast, sympathetic axons in the exocrine pancreas show no difference between individuals with or without T1D (102–104). Parasympathetic axons innervating the exocrine pancreas were also found decreased, but not the parasympathetic axons near the pancreatic islets in patients with recent onset T1D (105). It was reported that in addition to the decreased exocrine pancreas innervation and reduced pancreas volume, the cellular composition of the pancreas is altered in pre-symptomatic T1D (3, 4, 86). Therefore, it is possible that the exocrine insufficiency described in T1D could either contribute to or be a consequence of the neural decline due in part to the crosstalk between aPSCs and Schwann cells.

We acknowledge the bioinformatic nature of this study in which inferred interactions suggest pathways that may be of importance in the pathogenesis of T1D. Further research assessing protein interactions is necessary to validate these interactions. We also recognize that there is an inherent limitation of prior knowledge for inference, and CellChatDB, as well as other resources such as CellPhoneDB, ConnectomeDB, or LRdb have their own bias (106–109).

Nevertheless, this is the first study that analyzed cell-to-cell communication with scRNA transcriptomic data using CellChat to uncover the role of aPSC in the development and/or progression of T1D. We highlighted known and new signaling pathways such as the ANGPTL, CXCL, TGFB, NGF and PTN pathways. However, it is likely that the signaling pathways have greater value when viewed as a network rather than individual processes, and we should consider the crosstalk of different pathways to identify novel targets for therapeutic interventions. The changes in cellular communication found in nonendocrine cells, including aPSCs, support the concept of T1D as a disorder of the exocrine and endocrine pancreas.

## Conclusions

Analyzing intercellular communications, we have shown that aPSCs were among the main cells to undergo major changes in number and strength of cellular communication in T1D. We demonstrated that in T1D, aPSCs expressed higher levels of many ligands including ANGPTL4, CXCL12, TGFβ, NGF, FGF7 and ceased to secrete PTN. Identification of these novel changes in aPSCs cellular communication in T1D provides an insight into the potential role of aPSCs in the pathogenesis and progression of T1D that warrants further studies.

## Supplementary Material

Supplementary Files

This is a list of supplementary files associated with this preprint. Click to download.

- Additionalfile1.pdf
- Additionalfile2.xls
- Additionalfile3.tif
- Additionalfile5.tif
- Additionalfile4.tif
- VisualAbstract.tif

## Figures and Tables

**Table 1:** Aggregation of cell groups

Functional Categories	Cell groups
Endocrine	alpha, alpha-beta, cycling-alpha, beta, delta, gamma-epsilon
Exocrine	ductal, ductal-acinar, MUC5B-ductal, acinar
PSCs	aPSCs, qPSCs
Vascular	endothelial, pericytes
Immune	macrophages, mast cells
Schwann	Schwann cells

**Table 2:** Number of interactions among cell groups

ND control		Target						
		Endocrine	Exocrine	Immune	PSCs	Schwann	Vascular	Total
Source	Endocrine	27	61	5	15	5	57	170
	Exocrine	64	147	20	92	14	128	465
	Immune	0	2	2	3	0	5	12
	PSCs	21	36	6	18	6	23	110
	Schwann	20	40	5	18	4	25	112
	Vascular	18	58	6	29	6	48	165
	Total	150	344	44	175	35	286	
T1D		Target						
		Endocrine	Exocrine	Immune	PSCs	Schwann	Vascular	Total
Source	Endocrine	23	57	12	21	5	68	186
	Exocrine	62	180	42	103	26	140	553
	Immune	5	40	4	10	0	28	87
	PSCs	20	57	10	18	5	45	155
	Schwann	23	40	8	17	4	25	117
	Vascular	10	58	9	34	6	53	170
	Total	143	432	85	203	46	359	

**Table 3:** Direct signaling interaction from aPSCs to β-cells.

aPSC ligand	β-cell receptor	Probability	Probability pVal	Pathway name	Sample set
FGF2	FGFR1	0.00209	< 0.01	FGF	ND
PTN	SDC2	0.00064	< 0.01	PTN	ND
SLIT2	ROBO1	0.00083	< 0.01	SLIT	ND
SLIT2	ROBO2	0.00121	< 0.01	SLIT	ND
FGF2	FGFR1	0.00037	< 0.01	FGF	T1D
FGF7	FGFR1	0.00028	< 0.01	FGF	T1D
ANGPTL4	SDC2	0.00101	< 0.01	ANGPTL	T1D
MDK	SDC2	0.00076	< 0.01	MK	T1D
SLIT2	ROBO1	0.00153	< 0.01	SLIT	T1D
SLIT2	ROBO2	0.00214	< 0.01	SLIT	T1D

## Data Availability

The datasets analyzed during the current study are publicly available from the HPAP PANC-DB repository, (14–16).

## Abbreviations

T1D: type 1 diabetes
ND: non-diabetes
HPAP: Human Pancreas Analysis Program
nPOD: network for Pancreatic Organ Donors with Diabetes
PSC: pancreatic stellate cells
aPSCs: activated pancreatic stellate cells
qPSCs: quiescent pancreatic stellate cells
scRNA-seq: single cell RNA sequencing
snRNA-seq: single-nuclei RNA sequencing
UMAP: Uniform Manifold Approximation and Projection
SNN: Shared Nearest Neighbor
ANGPTL: angiopoietin-like protein
ANGPTL4: angiopoietin-like protein 4
SEMA3: semaphorin 3
PTN: pleiotrophin
FGF: fibroblast growth factor
IL1: interleukin 1
TGFB: transforming growth factorβ
ACVR1B: activin A receptor type 1B
ALK4: Activin receptor-like kinase 4
NGF: nerve growth factor
NGFR: nerve growth factor receptor
DEG: differential expressed genes
GSEA: Gene Set Enrichment Analysis
GO: Gene Ontology
KEGG: Kyoto Encyclopedia of Genes and Genomes
GSK-3β: glycogen synthase kinase 3β
LPL: lipoprotein lipase
PPAR: peroxisome proliferator-activated receptor
